# Hernia Active Living Trial (HALT): a feasibility study of a physical activity intervention for people with a bowel stoma who have a parastomal hernia/bulge

**DOI:** 10.1186/s40814-023-01329-8

**Published:** 2023-07-03

**Authors:** Julie Munro, William Goodman, Raymond Oliphant, Sarah Russell, Claire Taylor, Rebecca J. Beeken, Gill Hubbard

**Affiliations:** 1 Department of Nursing & Midwifery, Centre for Health Sciences, University of the Highlands and Islands, Inverness, UK; 2 Leeds Institute of Health Sciences, University of Leeds, Leeds, UK; 3 NHS Highland, Raigmore Hospital, Inverness, UK; 4 The Ostomy Studio, Wadhurst, East Sussex UK; 5 London North West University Healthcare NHS Trust & Visiting Lecturer, Chief Nursing Officer Macmillan Cancer saupport King’s College, London, UK

**Keywords:** Physical activity, Exercise, Stoma, Parastomal hernia, Feasibility

## Abstract

**Background:**

Parastomal bulging/hernia is a common complication associated with a stoma. Strengthening of the abdominal muscles via exercise may be a useful self-management strategy. The aim of this feasibility work was to address uncertainties around testing a Pilates-based exercise intervention for people with parastomal bulging.

**Methods:**

An exercise intervention was developed and tested in a single-arm trial (*n* = 17 recruited via social media) followed by a feasibility randomised controlled trial RCT (*n* = 19 recruited from hospitals). Adults with an ileostomy or colostomy with a bulge or diagnosed hernia around their stoma were eligible. The intervention involved a booklet, videos, and up to 12 online sessions with an exercise specialist. Feasibility outcomes included intervention acceptability, fidelity, adherence, and retention. Acceptability of self-report measures for quality of life, self-efficacy, and physical activity were assessed based on missing data within surveys pre- and post-intervention. Interviews (*n* = 12) explored participants’ qualitative experiences of the intervention.

**Results:**

Nineteen of 28 participants referred to the intervention completed the programme (67%) and received an average of 8 sessions, lasting a mean of 48 min. Sixteen participants completed follow-up measures (44% retention), with low levels of missing data across the different measures, apart from body image and work/social function quality of life subscales (50% and 56% missing, respectively). Themes from qualitative interviews related to the benefits of being involved, including behavioural and physical changes in addition to improved mental health. Identified barriers included time constraints and health issues.

**Conclusions:**

The exercise intervention was feasible to deliver, acceptable to participants, and potentially helpful. Qualitative data suggests physical and psycholosical benefits. Strategies to improve retention need to be included in a future study.

**Trial registration:**

ISRCTN, ISRCTN15207595. Registered on 11 July 2019

## Key messages regarding feasibility

- What uncertainties existed regarding the feasibility?
  An exercise intervention for people living with a parastomal hernia/bulge had not been conducted previously. Therefore, there were uncertainties regarding the safety of the intervention, its acceptability to the participants, whether it would be conducted as intended, and if participants would adhere to this.
- What are the key feasibility findings?
  We found that the exercise intervention was safe, it was conducted as intended, participants adhered to the intervention, and it was acceptable to them. However, we had issues with the retention of participants and missing data on certain variables.
- What are the implications of the feasibility findings for the design of the main study?
  Strategies for the retention of participants need to be devised to address this issue and consideration needs to be given to the scales used for quality of life due to missing data.

## Background

A stoma is an artificial opening on the surface of the abdomen that has been surgically created in order to divert the flow of faeces or urine. In Europe, approximately 700,000 people are living with a stoma, and in the USA, more than 1 million people have a stoma [1]. Within the UK, estimates suggest that just under 11,500 patients diagnosed with rectal cancer [2] and around 2000 people with inflammatory bowel disease have a stoma formed each year [3]. Parastomal hernia (PSH) and parastomal bulging is a common late stomal problem [4], with prevalence estimates over 30% by 12 months, 40% by 2 years, and 50% or higher at a longer duration of follow-up [5]. A parastomal hernia (PSH) is defined by the European Hernia Society as ‘an abnormal protrusion of the contents of the abdominal cavity through the abdominal wall defect created during placement of a colostomy, ileostomy, or ileal conduit stoma [6]. A parastomal bulge is similarly defined but does not include a formal diagnosis [6]. It is difficult to differentiate between the two clinically [7], and from a patient perspective, lived experience of PSH and parastomal bulge may be indistinguishable.

Quality of life (QOL) can be considered a multidimensional construct that can help to capture an individual’s view on their experience of health and allows for the evaluation of interventions designed to improve this [8, 9]. Few studies have specifically explored the quality of life in people with a parastomal bulge or PSH. Two cross-sectional studies from Sweden (*n* = 70) [10] and Denmark (*n* = 1265) [11] found that QOL was worse in those with a parastomal bulge compared to those without a parastomal bulge. Furthermore, the study from Denmark also reported that QOL scores for patients with a large parastomal bulge (> 10 cm) were significantly worse than for patients with a small bulge [11]. Lower QOL associated with the size of the bulge may be related to body image concerns [12] as a cross-sectional study found that patients with colorectal cancer and a stoma (*n* = 35) who had body image concerns also reported lower QOL [13]. Non-surgical management of PSH is considered a research priority for this patient population [14].

Central to developing self-management approaches to support improved QOL for this group is understanding the aetiology of parastomal bulging. However, there is a paucity of prospective data about the natural history and trajectory of parastomal bulging and whether parastomal bulging severity progression can be arrested [15, 16]. Following abdominal surgery, the physiology of the abdominal wall is altered with potential damage to nerve supply and atrophy of the midline muscular wall [17]. Surgery for creating a stoma alters the physiology in the same way and creates a further site of weakness by leaving a hole in the abdominal wall. Evidence indicates that there is muscular atrophy directly below the stoma site, resulting in a change of forces and pressure on the abdominal wall [18]. One hypothesis is that abdominal exercises could counteract the weakness as a result of surgery and stoma creation [19, 20].

Another PSH management theory is that abdominal and breathing exercises contribute towards strengthening the body core so that there is better control of intra-abdominal pressure. The Association of Stoma Care Nurses highlights intra-abdominal pressure as a risk factor for PSH [21] based on expert opinion. Further research and data are needed to confirm this aetiology in this patient group. Studies have highlighted a trend towards inactivity after stoma formation surgery, with fear of PSH being a major deterrent to exercise [22–24]. A decrease in physical activity also increases the risk of cancer recurrence in this population [25].

This study assessed the feasibility of an exercise programme for people with a parastomal bulge. Large-scale, statistically powered randomised controlled trials (RCTs) are increasingly informed by one or more feasibility studies that are designed to provide evidence in order to make informed decisions about whether an intervention and the research methods can be replicated in a larger multi-centre study [26]. By assessing the feasibility outcomes and with the discussion with a Steering Group, recommendations on whether it is appropriate to proceed to an effectiveness RCT will be established, or if further work is necessary to further refine the intervention and methods [27].

## Aims and objectives

The aim of a future effectiveness RCT is to determine whether a structured exercise intervention improves QOL for people living with a parastomal bulge. The aim of this feasibility study was to address uncertainties relating to exercise intervention and trial methods. The objectives were as follows:

1. To determine intervention fidelity
2. To determine intervention adherence
3. To determine intervention acceptability and safety
4. To determine eligible patients’ consent rate
5. To determine participants’ acceptability of RCT design (retention and missing data rates)
6. To determine participants’ acceptability of outcome measures

## Methods

The feasibility study was conducted in line with a published a priori protocol [28] and statistical analysis plans [29]. Some changes to the study were made because of the COVID-19 pandemic (Appendix 1).

### Single arm design

The single-arm trial consented 17 participants before progression to the feasibility RCT. Further details are available in Appendix 2.

### Design

In this feasibility study, an exercise intervention was developed and piloted in a single-arm trial followed by a feasibility RCT.

### Participants

#### Eligibility criteria for participants

Adults 16 years + , ≥ 3 months post-stoma formation surgery for bowel disease (e.g. inflammatory bowel disease, colorectal cancer), with a colostomy or ileostomy, with a self-assessed parastomal bulge or with a clinical diagnosis of a PSH were eligible.

People who were already doing core training (e.g. Pilates, yoga) were excluded. People who did not have access to the Internet were excluded because the intervention was delivered online by video.

#### Recruitment

Feasibility RCT patients were recruited from 2 trial sites. Patient recruitment from a large metropolitan teaching hospital was conducted by a research nurse. Patient recruitment from an acute district hospital was conducted by a consultant colorectal surgeon who completed screening for eligible patients from their outpatient list and sent out invitation letters to potentially eligible patients. Interested participants were directed to contact a research assistant directly by email or telephone.

#### Data collection

Three methods were used to collect data from participants—questionnaire, diary, and semi-structured interview. Participants completed an online questionnaire and exercise diary that they accessed via the Internet from their own homes. The questionnaire was completed at baseline and follow-up, and the diary was completed each week of the 12-week exercise programme. At follow-up, a research assistant (JM, WG) conducted a semi-structured interview with participants by telephone or video conference.

### Intervention

The exercise intervention was developed by the research team and the Patient Advisory Group via online meetings and email exchanges. This intervention drew on the core principles of self-determination theory (SDT); these include conditions that support a person’s basic psychological needs (their need for autonomy, competence, and relatedness) and foster the most volitional and intrinsic forms of motivation for initiation and long-term maintenance of exercise [30].

The exercise intervention had three core components:

1. Exercise booklet sent by email to all participants. The exercises illustrated and described in the booklet were based on the Australian Physiotherapy and Pilates Institute methods programme [31], and the booklet included a hyperlink to a YouTube channel that showed each of the exercises.
2. Exercise videos available on a private YouTube channel. In each video, a member of the Patient Advisory Group performed each exercise whilst being given verbal instructions by a clinical exercise instructor. There were 13 exercise videos in total.
3. Exercise sessions delivered online by a clinical exercise instructor. In this feasibility study, the intervention was delivered separately by two clinical exercise instructors who both held a Register of Exercise Professional Level 4 cancer rehabilitation qualification and had previously supported clinical populations, including people with a stoma, to engage in exercise, including Pilates. Participants could arrange to meet once a week online over a period of 12 weeks (if required) for 15–45 min with an instructor.

Depending on participant ability, safety, and time commitments, participants could use the booklet and/or watch the exercise videos and/or have a one-to-one exercise session with a clinical exercise instructor. There was in-built flexibility so that the intervention could be tailored to address the unique needs of each participant.

### Control group

Participants randomised to the control group were signposted with information and guidance about physical activity from UK Charities Ileostomy and Internal Pouch Association, and Colostomy UK.

### Outcomes

The main outcome of the feasibility study was establishing the fidelity, adherence, acceptability, and safety of the proposed intervention. This led to a consultation with an Independent Steering Group to proceed or not to an effectiveness RCT using the following traffic light system to guide decision-making (Table 1). Members of this group included a clinical exercise specialist, a colorectal consultant, and an expert in trial design and evaluation. The group met with the research team and funder representatives in a 2-h meeting to discuss the study findings.

**Table 1:**
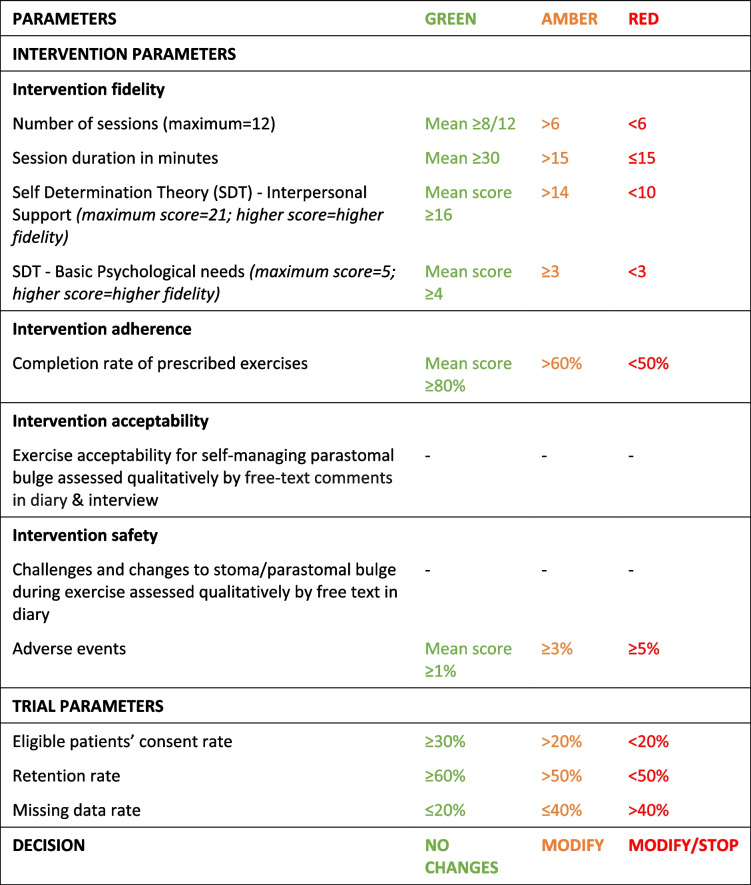
Criteria for progression to an effectiveness RCT

The outcomes used to assess the feasibility and acceptability of the intervention were as follows.

#### Intervention fidelity

Intervention fidelity was defined as the extent to which the intervention was delivered as intended. Quantitative measures of fidelity were the number of consultations delivered by the instructor and duration, which based on our previous physical activity intervention we estimated to be an average of 10 consultations over a 12-week programme and 35 min [32]. Two instruments were used to assess if the intervention was delivered in line with the principles of self-determination theory:

1. Four online consultations were recorded with participants’ permission and two researchers (GH, CT) assessed these using the Interpersonal Support in Physical Activity Observational Tool [33]. A total score was summed, with a higher score indicating higher intervention fidelity.
2. Participants receiving the intervention completed subscales on competence and autonomy from the Basic Psychological Needs in Exercise Scale which were relevant to the intervention.

#### Intervention adherence

Intervention adherence was defined as the completion rate of the prescribed exercises by participants. Participants used the online diary to record each week the extent to which they completed the exercises prescribed by the instructor. There were five response options: all of it 100%, most of it (75%), about half of it (50%), some of it (25%), and none of it (0%).

#### Intervention acceptability

The acceptability of the intervention was investigated by free-text comments in the diary and during a semi-structured interview. Participants were given the opportunity to comment in the diary on how they felt doing the exercises.

#### Intervention safety

Participants used the diary to report any challenges or any changes with their stoma/parastomal bulge during their participation in the exercise programme. Serious adverse events (AEs) were reported as part of the ethical conduct of the study. A standard SAE form was completed by the clinical exercise instructors if required.

The outcomes used to assess the feasibility and acceptability of trial parameters were as follows:

#### Eligible patients’ consent rate

Eligible patients’ consent rate was defined as the number of patients who enquired about the study, who then went on to consent to participate. This includes participants from social media (single arm) and hospital recruitment (feasibility RCT). Eligibility was confirmed by the research team.

#### Acceptability of RCT design

Eligible patients’ consent rate and retention rate were used as proxy measures of the acceptability of the RCT design. The retention rate was defined as the number of consenting participants who completed baseline and follow-up measures. Based on our previous study of a physical activity intervention for people with stoma, we estimated a 60% retention rate [32].

#### Acceptability and data availability of outcome measures

The acceptability of instruments to measure outcomes was explored in the semi-structured interviews conducted with participants at the end of the study. Data availability refers to the amount of data available for analyses. In this feasibility study, we therefore assessed the amount of complete data for the following outcomes:

##### Quality of life

The EQ-5D was used as a generic measure of QOL. Stoma-related QOL was measured using the Stoma-QOL [34]. It is a 21-item questionnaire, 19 items covering the 5 domains of work/social functioning, sexual/body image, stoma function, financial concerns, and skin irritation. *Body image:* The Body Image Scale [35] was used for assessing body image. This is a 10-item instrument previously validated with ostomy patients [36].

##### Physical functioning

The Patient-Specific Functional Scale (PSFS) focuses on the patient’s opinion of their function in order to provide clinicians with a reliable and valid self-reported outcome measure [37]. Participants list up to five activities that are limited by their condition (their PSH). It is used in clinical practice and research to assess if there is a meaningful change in functional status that has occurred over time [37].

##### Self-efficacy

The Exercise Regularly Scale [38] was adapted to assess self-efficacy. There were four items. All items were rated from 1 (not at all confident) to 10 (totally confident).

##### Physical activity

This was measured through 4 self-report questions asking how many times in the past week participants had been physically active inside and outside the home and typical duration in minutes. This measure was adapted from a single-item physical activity measure [39].

Additional questions related to the individual’s parastomal bulging were also asked, these included questions of size, pain, self-management, body image, and restrictions to daily activities.

A list of all the survey questions can be accessed in Appendix 3.

### Sample size

This study assessed the feasibility of recruiting people with a parastomal bulge in a large teaching hospital primarily serving a large urban population, a general hospital serving a small urban and remote and rural population, and via social media. These recruitment methods would yield a representative target population for the planned future RCT. In the future RCT, we intend for QOL to be our primary outcome, and in line with Whitehead et al., a sample size of 20 was considered appropriate [40].

### Randomisation

Participants for the feasibility RCT were randomly allocated to intervention or control groups by a research assistant using MinimPy [41] which is a free randomisation software package to manage the process of minimising the difference among trial groups with respect to pre-selected categorical factors, i.e. site. Participants were randomised on a 2:1 basis, intervention to control. The research team was responsible for enrolling participants into the allocated group and referral to the intervention or providing the control group materials as specified.

### Data analysis

Data were analysed using SPSS v26. The rates of eligibility, retention, and follow-up were reported as percentages, as were the missing data rates for each outcome of interest. The means and standard deviations were calculated and presented. Change scores from baseline to follow-up were calculated and an independent sample *t*-test was conducted with the grouping variable being the control or intervention condition. Due to the small numbers in this study and the objectives being primarily feasibility, only 95% confidence intervals are reported.

Participants were asked to record their weekly exercise in an anonymous online diary. They recorded the level of exercises they had been asked to work at, how many days a week the exercises had been prescribed, how long they should have been exercising for, and how much of the prescribed activity they had completed. These data were collated and are presented with frequencies and percentages. Qualitative thematic analyses of audio-recorded interviews and focus groups were conducted using the framework approach [42] by JM and WG with checking conducted by GH.

## Patient and public involvement

The Patient Advisory Group (PAG) was led by the public involvement lead at Bowel Research UK. Two members of the PAG attended all research team meetings to contribute to the management or running of the study. The PAG provided feedback about the content of the intervention and about the lay language being used in public-facing documentation (e.g. patient information sheet and Social media advert). The PAG also shared their range of expertise and designed the HALT trial logo and participant information sheets, produced artwork to illustrate the exercises within the exercise booklet, acted as demonstrators for our exercise videos, and were on hand for any queries to help develop the project. This ensured patients were at the core of the work.

## Results

### Consent and retention rates

**Fig. 1:**
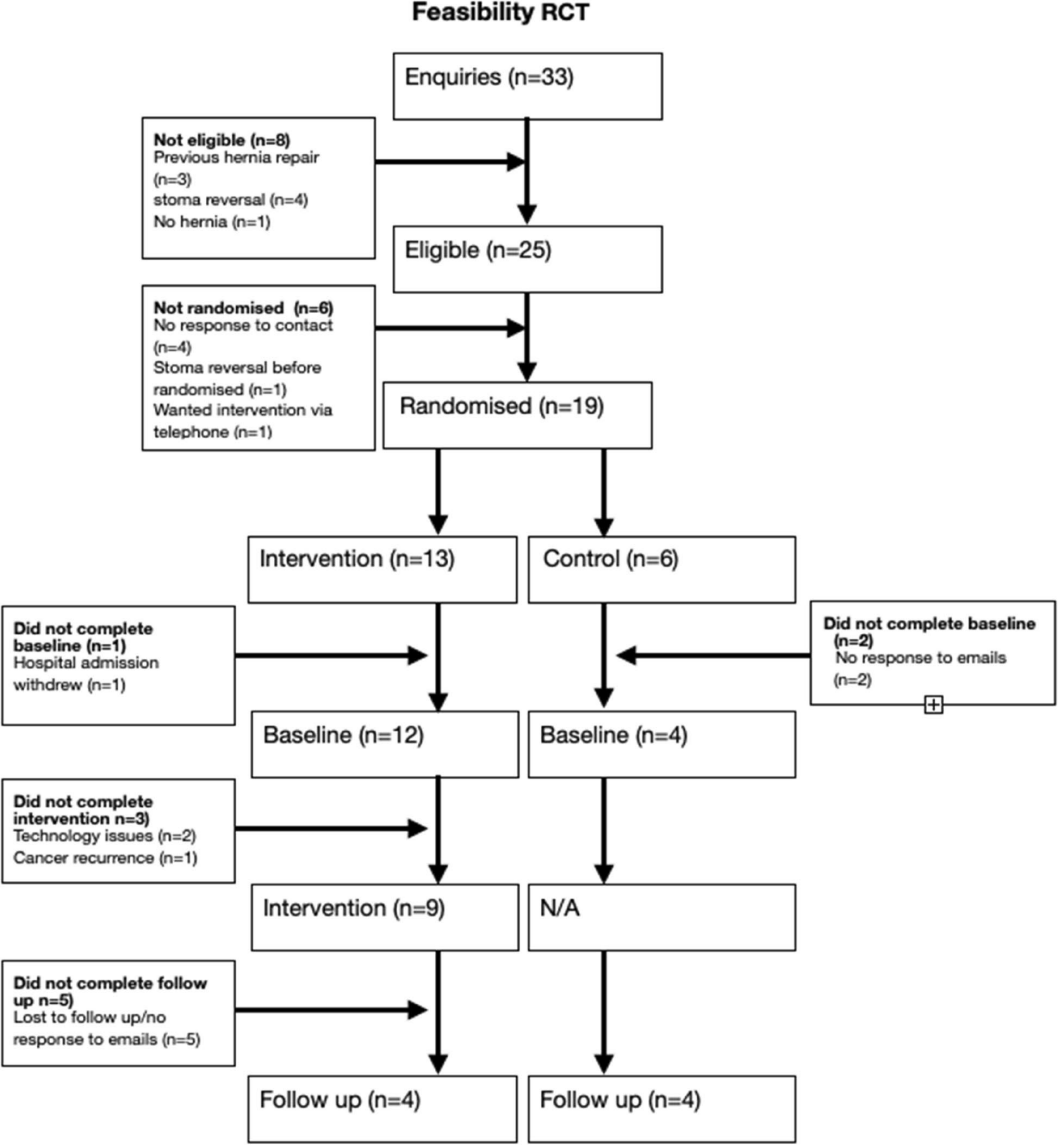
Participant recruitment flowchart

Figure 1 displays the recruitment flowchart for the feasibility RCT.

Thirty-three people enquired about the study over a period of 13 weeks, of which 25 (76%) were eligible to take part, and 19 (76% eligible patient consent rate) were randomised to the intervention or control group. Sixteen participants completed baseline measures, and 8 completed follow-up measures, a 42% retention rate. Of the 13 intervention referrals, 9 (69%) participants completed the intervention.

### Participant characteristics

Characteristics are summarised in Table 2.

**Table 2:** Baseline characteristics of participants

**Variable**	**Hospital, *n* = 19 (%)**
**Gender**	
Male	11 (58%)
Female	8 (42%)
**Age (years)**	Mean 64 (min 39: max 75)
**Diagnosis**	
Bowel cancer	10 (53%)
Crohn’s	0
Diverticulitis	2 (11%)
Ulcerative colitis	5 (26%)
Others (including injury, radiation damage, abscesses)	2 (11%)
**Type of stoma**	
Colostomy	10 (53%)
Ileostomy	9 (47%)

Eleven (58%) participants were male, the age range was 39–75, 10 (53%) were diagnosed with bowel cancer, and 10 had a colostomy (53%).

Appendix 2 details the characteristics of the single-arm participants and combined with the RCT data.

### Missing data rate

The majority of the outcome measures were well completed by the participants (missing data ≤ 20%). However, certain subscales for the Stoma-QOL measure showed high rates of missing data, for example, work/social function (control group = 100% and intervention group = 50%) and sexuality/body image (control group = 75% and intervention group = 50%). There was also a high level of missing data for the control group on the question of ever having considered surgical repair for their bulge/hernia (50%). A complete breakdown of the rate of missing data for the outcome measures can be found in Appendix 4.

The feasibility study was not powered to detect statistically significant changes in the outcome measures. However, it is useful to see the data to assess if the distribution of responses were within a typical range [43, 44]. The data can be found in Appendix 5 and show that baseline and follow-up distributions were within a typical range for the EQ-5D descriptive score.

### Intervention fidelity

The maximum number of exercise sessions available to participants was 12. Table 3 shows that participants received on average 8 sessions, lasting on average 48 min. Feedback from the exercise instructors was that some participants did not require more than 8 sessions and therefore did not continue after the eighth session.

**Table 3:** Average number of sessions and duration of participant intervention

	Mean	Median	Range
Average number of sessions per participant	8	7	5–12
Average duration of session [min]	48	48	31–62.5

The clinical exercise instructors delivered the exercise sessions in accordance with the principles of SDT. Both researchers (GH, CT) gave a maximum score of 21 for three video exercise sessions using the Interpersonal Support in Physical Activity Observational Tool. One researcher scored the fourth video session as 21 and the other researcher gave a score of 16. The total score was therefore 163 out of 168, giving an average score of 20.3.

The data for the Basic Psychological Needs in Exercise Scale (range 1–5) found that for the competence subscale mean scores increased from 2.98 (SD: 1.02) to 3.26 (SD: 0.88) and for the autonomy subscale mean scores increased from 2.95 (SD: 1.03) to 3.44 (SD: 1.17) from baseline to follow-up.

### Intervention adherence

Data taken from participant’s online diaries shows how much of the prescribed exercise participants completed on any given week. The online diary was used by 15 participants. Ninety-two per cent of the exercises prescribed were completed (completion was defined as > 75% of the exercise prescription given).

### Intervention safety

No serious adverse events were reported.

### Qualitative interviews

Twelve interviews were conducted with participants. Thematic analysis was completed by three researchers (WG; JM; GH).

#### Reasons for being involved

The majority of participants decided to sign up for the trial to help them self-manage their parastomal bulge:

*“If I can find a way of managing that hernia so it doesn’t get any worse in a more proactive way” (ID02) M*

One participant joined the trial with the intention of improving her abdominal control in the hope of avoiding surgery:

*“I’m told it will be a huge thing if I do have surgery, and the chances of the hernia coming back on the other side is phenomenally high, so, you know, all in all, I don’t want that, I don’t want to go down that route at all. So, the idea of tightening things up with a view to making things better, really, or less chance of needing any more surgery was all to the good” (ID08) F*

#### Physical changes

Participants who received the exercise intervention perceived physical improvements such as reducing the size of hernia, weight loss, core strengthening, core control, improved posture, and less need for support garments due to better core control:

*“I feel like more stable when I’m running and that sort of thing, so, like, my core just feels stronger” (ID02) M*

*“I wish I’d taken photographs before but I didn’t, it is definitely smaller, the hernia… I could feel everything was tightening up, and for somebody of my age, that is quite amazing, really. The actual reduction in the hernia has probably been certainly more gradual. I mean, it wasn’t, you know, one day it was there and the next day it wasn’t sort of thing, it’s not that dramatic” (ID07) F*

*“... before I started doing the course with [name of exercise instructor], I would need to wear it [support garment] all the time, it became really uncomfortable….Now when I’m sat here it contains itself….I’m keeping my core a little bit more tense, I’m holding it together, and it’s sort of self-perpetuating” (ID29) M*

Physical changes were also described by participants in the diary about any challenges and issues that they experienced relating to their stoma and parastomal bulge during the study. Select comments are presented in Table 4.

**Table 4:** Participant written comments about physical change

**ID**	**Themes and written comments**
**Change to the hernia**	
29	Overnight ……..my hernia reduced considerably. Since then normal movement does bring it back out, but only half the size it was before. The reduced size is much more comfortable. Occurred overnight, but has persisted so far (3 days). M
02	I am feeling really good and notice they are making a positive difference to my bulge the day after I do them. Also, now when sneezing I can feel a subconscious movement to “tighten the belt” which supports the area. M
**Strength, posture, and tone**	
14	Can feel a difference in my abdominal muscles, particularly around the hernia. Feels stronger, and my posture is much better M
14	I feel the best I have for 10 years. My abdominal muscles feel stronger, my posture is much better, and my hernia feels tighter. I am enjoying it immensely, this will become a way of life. M
14	The area around my hernia already feels more toned, probably because I haven’t used those muscles for 44 years! M
5	I have never had any issues with my hernia whilst doing these exercises… I now have developed muscle control of my tummy, and now have a much more toned body because of the exercises…I will be continuing these exercises to help improve my core, and general wellbeing. F
02	I am feeling stronger and more conditioned. M
**Abdominal control**	
11	Feeling the benefit of all the exercises that I am doing, feeling more in control of the muscles in my abdomen, no issues with my hernia at all. F
11	I felt that I have really benefited from doing all the exercises, feeling that I now have a hold of my pelvic floor and improved greatly my core muscles. F
02	Got a much better core connection without my ribs lifting due to the slight modifications. M

#### Clinical exercise specialists

The feedback given about the expertise and support from the two clinical exercise specialists who delivered the exercise intervention was unanimously positive. They provided a non-judgemental environment and participants felt that the positivity and attention given to them at each session was ‘first class’ and that despite sessions being via video call they felt very personal and there was close attention to detail:

*“[name of exercise instructor] made it so easy to do, sort of face-to-face online, if that makes sense, that you felt she was in the room with you when you were doing stuff.” (ID03) F*

*“But, yeah, what struck me was the attention to detail that even remote video classes can have in terms of you’ve got the camera positioned correctly then it really enabled some pretty first-class feedback” (ID07) F*

#### Behaviour change

Participants shared a number of ways they had changed their behaviour and way of thinking about exercise. The intervention encouraged them to think about their physical activity levels and had gained confidence to things they may have previously avoided. Sub-conscious changes to breathing habits and automatically engaging their core muscles before a daily activity indicate that the intervention has made some positive behaviour changes:

*“I actually do much bigger walks……my son can benefit from me being able to do things with him like this now without it being a case of ‘Oh, I’m so tired, no, no, this is going to hurt, I can’t do this’, it’s like ‘Well, give it a go’” (ID03) F*

*“This has really brought home to me the importance of doing the behind the scenes things before you go off and do the thing that you perhaps enjoy doing most…...having sort of recognised more what it is, it’s easier to… to sort of consciously, almost subconsciously, engage now than it would have been before I started.” (ID12) F*

#### Confidence

Many participants mentioned the improvement in their confidence to move more and be in control of their health and health-related behaviours. They felt more equipped with the skills to breathe better, engage their muscles and be more body confident:

*“The biggest thing has been my change in attitude towards my stoma. It’s no longer a negative thing for me… I control it, it doesn’t control me anymore” (ID03) F*

*“The body confidence, I think, yeah, it’s helped me with that, and it’s the idea of getting into the best shape I can get into for me is a healthy one. So, I’m not punishing myself, but rather I’m actually going down a very healthy path of thinking actually this is really good for me now and I’m not being ill every day, I’m feeling pretty good” (ID11) F*

#### Mental health

Several participants referred to their mental health. The intervention gave one participant enough confidence and change in mindset to get back to work where previously they felt the workplace would not want them, and others report changes in their mental health and state the intervention as a life-changing experience:

*“I’m genuinely starting now to get my CV together ready to go back to work, which before the trial, I would have been thinking twice about, because the discomfort and the pain and the tiredness were making me kind of go “Actually, what employer is going to really want me?’ To now going ‘You know what, yeah, there’s issues, but I know how to manage them, and I will do…’” (ID03) F*

*“I’d go so far as to say it’s a life changing experience, I’m absolutely chuffed, and it’s… yeah, to think something so… so simple can be so effective” (ID07) F*

#### Barriers

Participants noted a few common barriers to successfully following the exercise prescription each week. Health issues were the main barrier because they had an effect on motivation. Other participants touched on time commitments, and their own preconceptions setting them back such as anxieties about not being able to do what was asked or fear of judgement. These barriers dissipated after meeting with the clinical exercise specialists.

*“I work from home and, you know, I was at meetings a lot, they pop up whenever they want to. That was the only difficulty, was trying to find a time where… where, like, in the day you’re guaranteed that you have half an hour” (ID02) M*

*“I think the biggest barrier was I was a little bit worried about doing it… You know, sometimes you kind of think “they’re going to think I can do things that I can’t do.” And that came very much from me because I was annoyed that I couldn’t do what I wanted to do” (ID03) F*

#### Intervention content

##### Technology

Participants reported that online video conferencing worked extremely well, and the exercise sessions were as good as being delivered in person. It was also noted that the online experience gave more flexibility, saved travel time, allowed them to be anywhere, and kept them safe during the COVID-19 pandemic. There were issues with poor connections on occasion, and not feeling *tech savvy*, but these were navigated successfully.

*“And I’ve got to say that a virtual class, I think, is as effective as physically being in someone’s studio, which of course isn’t possible at the moment” (ID03) F*

*“In fact, personally, I found quite a lot of these things better than if you’d have to go somewhere to meet somebody to do it in person, because that usually involves quite a lot of wasting time, you know, you’ve got to travel to somewhere, find somewhere to park, then you might have to be sitting in a waiting room for goodness knows how long, and all that sort of thing. So, for me, doing it this way on Zoom is much, much better, I much prefer it” (ID10) F*

#### Exercises

Feedback about the type, style, and difficulty level of the exercises was positive. On the whole, participants found the exercises manageable but challenging to begin with, then gained confidence and improved over time with the support of the clinical exercise specialist. The importance of technique and visual feedback along with advice from the exercise specialist was highlighted:

*“They [the exercises] look easy, but when you’ve held it for however long suddenly it’s not so easy anymore. And it was just, I imagined they’d be much… I imagined they’d be much easier than they were at first” (ID19). M*

*“I started off obviously doing the very basic ones, the pelvic tilt and the core and everything, and for me that was a revelation because I’ve never done anything like that before, and I can actually feel the difference quite quickly actually within a couple of weeks” (ID13) F*

### Results from the advisory steering group

A summary of the results against a priori criteria is presented in Table 5. One recommendation from the steering group was to describe participants’ improvements in the different exercises, e.g. if they moved from level 1 to level 3 during the programme. However, the main concern was the low retention rate. Strategies to improve this were suggested including clearer expectations of completion of follow-up surveys and building a good relationship through more regular contact such as regular text messaging. Another issue raised was the need to describe participants in greater detail in order to assess whether specific groups of patients would benefit from the intervention were it to be implemented in practice. These more detailed participant characteristics were missing from the feasibility trial.

**Table 5:**
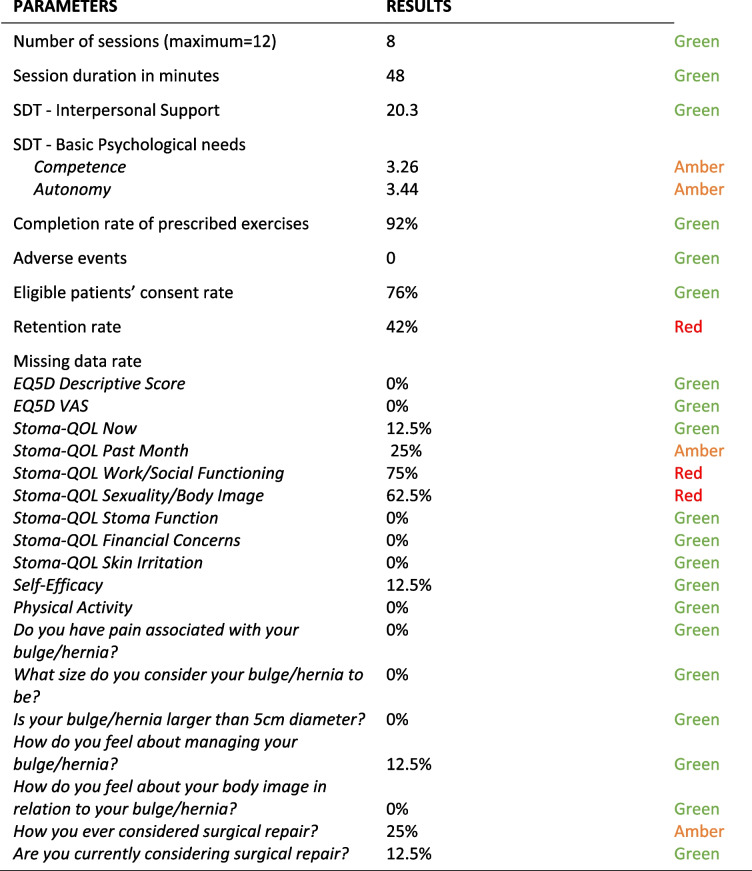
Summary of the results against a priori criteria

The steering group concluded that these issues could be addressed and that a full trial of the intervention should proceed but with clear strategies included for improving the retention rate. The full trial should also collect more detailed sociodemographic information from participants.

## Discussion

This feasibility study suggests that a Pilates-based exercise programme for people with a stoma and parastomal bulging is safe, acceptable, and feasible to conduct. However, a couple of points were highlighted by the trial steering group, retention of participants is an issue that needs to be addressed before proceeding with a full trial and a greater understanding of participant characteristics would be useful to assess a future trial. Despite this issue, the recommendation from the steering group was that we should proceed to a full trial of this exercise programme.

Data for recruitment, consent, and retention of participants are important for assessing the feasibility of a full trial. The eligible consent rate was similar in recruiting participants via social media and hospitals. It was also feasible to recruit participants across a range of age groups, gender, bowel disease, and type of stoma. The retention rate was slightly higher in participants recruited via social media than hospital which may reflect a higher level of familiarity and comfort with online interactions. It is not clear why participants did not complete measures at follow-up which makes it difficult to know which strategies could be used in a future RCT to improve the retention rate. A recent systematic review and meta-analyses of strategies to improve retention in randomised trials concluded that there is no high-certainty evidence pointing to an effective strategy but did find moderate-certainty evidence for monetary reward [45]. The trial steering group also recommended building in clearer expectations for participants as to what was expected at the end of the exercise programme to ensure they are aware of the follow-up measures. Furthermore, check-up messages sent by the research team during the 12-week programme were suggested to maintain contact with the participants during this period.

Another key objective of the feasibility study was to assess the missing data from the outcome measures. The proportion of missing data from the majority of the outcome measures was small to none suggesting that participants did not have any trouble with the questions that we asked them. However, missing data for two subscales of the Stoma-QOL scale (work/social functioning and sexuality/body image) was substantial; this could suggest that these measures are not fit for the sample we are recruiting. On the other hand, the items on the work/social functioning subscale may not have been applicable due to participants completing these during national lockdowns in the UK and if participants were furloughed. Both of these subscales should be reviewed by a PAG, with alternative scales considered before proceeding with a future RCT.

The exercise intervention that we developed was delivered as intended and in accordance with SDT principles. All three intervention components were acceptable to participants with the one-to-one sessions singled out by all participants as being particularly helpful. There was variation in the number and duration of sessions delivered which can be attributed to different needs for support from an exercise instructor, with some participants requiring additional support about the use of video conferencing technology suggesting the need for a walkthrough guide for those that are not tech-savvy. However, adherence to the prescribed exercises was high with participants perceiving physical and mental health benefits to taking part in the exercise intervention. Physical benefits aligned with the hypothesised benefits of the exercise programme, i.e. improved breathing technique, core control, and strength. Furthermore, as the steering group highlighted, we need a greater understanding of the characteristics of participants who take part in the trial to ensure that the intervention is effective across the breadth of the stoma community. These relevant variables could include ethnicity, level of education, and employment, as well as Internet use which may be important as a recent systematic review has indicated that digital physical activity interventions could be less effective for those from low socioeconomic status groups [46].

Furthermore, the exercise programme was safe as there were no adverse events. Participants experienced challenges and issues during the exercise programme including pain and discomfort around the stoma, but these were within acceptable limits and not all were attributed to the exercise programme. The distribution of responses for outcome measures was also within a typical range [43, 44], suggesting that the intervention and trial procedures did not have a negative impact upon participants.

## Conclusions

The exercise intervention is feasible to deliver and acceptable to participants. In a future study, more information about participants’ characteristics is required in order to assess if the study and the intervention if implemented in practice, would attract a range of patients who it is designed to benefit. Additional information on, for example, ethnicity, level of education, employment, and Internet use may be relevant. Strategies to improve retention need to be included in future effectiveness RCT.

## Figures and Tables

**Fig. 2:**
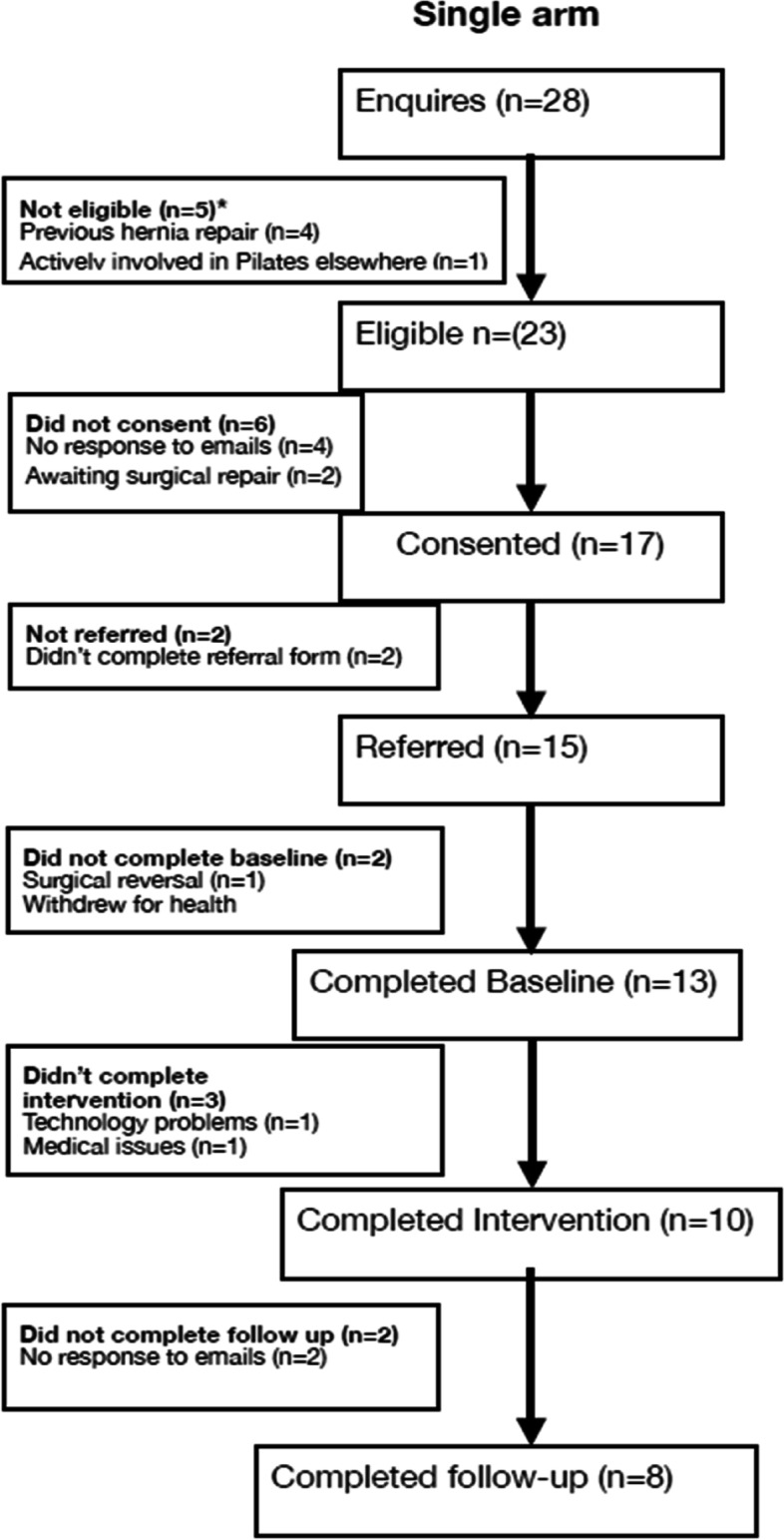
Single-arm recruitment via social media

## Data Availability

The intervention materials, questionnaires, PIS, consent form, and an anonymised dataset are available from the principal investigator upon reasonable request.

## Appendix 1

### Change to protocol

Changes made to the published protocol [28] due to the COVID-19 pandemic.

#### Recruitment

All recruitment in hospitals was ceased, and we reverted to recruitment by social media only in the single-arm trial during the first waves of the pandemic. Recruitment in the hospital was allowed by the NHS director in research into the feasibility RCT.

#### Intervention

Two changes were made to the intervention:

1. In the interests of safety, all exercise sessions delivered by the clinical exercise instructor were delivered online. That is, participants were not given the option of having the session conducted in-person as originally intended.
2. In the first wave of the UK pandemic, there were concerns about transmission of the coronavirus on parcels delivered to the home. Hence, in the interests of safety and as a precaution, we did not post out to participants a biofeedback stabiliser to help them monitor intraabdominal pressure during exercise.

#### Measuring intervention adherence and safety

Instead of providing a paper version of an exercise diary, this was moved to online software as it was considered high risk during the first waves of the pandemic to post items out to participants.

#### Patient-reported outcomes

It was necessary to remove some outcome measures to avoid the use of NHS staff during the pandemic and to avoid the risk of viral infection from parcels. Hence, in the interests of safety and as a precaution, hernia classification, muscle activation, body composition, and accelerometer-measured physical activity were not measured.

#### Eligibility

Following the initial ethical approval and after discussion with clinical teams on both sites, it was decided that the exclusion of patients who had undergone a previous hernia repair was not required. This patient group has a high risk of hernia recurrence and would benefit from the intervention in the same way as those with an existing parastomal bulge. The approval for this amendment was delayed due to the COVID-19 pandemic. The approval to include patients with a previous hernia repair came with only 2 weeks remaining of recruitment and therefore did not involve any new patients who had undergone a repair.

## Appendix 2

### Single-arm summary data

Single-arm trial for intervention development (social media recruitment). An advertisement was disseminated by the members of the Patient Advisory Group and by relevant stoma charities (Ileostomy and Internal Pouch Association, Colostomy UK) on both Facebook and Twitter. Interested participants contacted the research team directly.

The single-arm trial recruited participants through social media, 28 people enquired about the study over a period of 5 days, of which 23 (82%) were eligible to take part. Seventeen eligible participants consented to the study, a 74% eligible patient consent rate, and of these, 15 were referred to the clinical exercise specialist. Thirteen participants completed baseline measures, and 8 of these completed follow-up measures, with a 47% retention rate. Of the 15 intervention referrals, 10 (66%) participants completed the intervention.

Recruitment details can be seen in Fig. 2.

**Table 6:** Baseline characteristics of participants

**Variable**	**Total sample, ** ***n*** ** = 36 (%)**	**Social media, ** ***n*** ** = 17 (%)**	**Hospital, ** ***n*** ** = 19 (%)**
**Gender**			
Male	21 (58%)	10 (59%)	11 (58%)
Female	15 (42%)	7 (41%)	8 (42%)
**Age (years)**	Mean 58 (min 25: max 75)	Mean 54 (min 25: max 71)	Mean 64 (min 39: max 75)
**Diagnosis**			
Bowel cancer	12 (33%)	2 (12%)	10 (53%)
Crohn’s	1 (3%)	1 (6%)	0
Diverticulitis	7 (19%)	5 (29%)	2 (11%)
Ulcerative colitis	7 (19%)	2 (12%)	5 (26%)
Others (including injury, radiation damage, abscesses)	9 (25%)	7 (41%)	2 (11%)
**Type of stoma**			
Colostomy	20 (56%)	10 (56%)	10 (53%)
Ileostomy	16 (44%)	7 (44%)	9 (47%)

## Appendix 3

### Survey questionnaire

Please enter the study ID you have been given by the researcher.

#### Quality of life questions

Under each heading, please choose ONE box that best describes your health Today.

Mobility:

*I have no problems in walking about.*

*I have slight problems in walking about.*

*I have moderate problems in walking about.*

*I have severe problems in walking about.*

*I am unable to walk about.*

Self-care:

*I have no problems washing or dressing myself.*

*I have slight problems washing or dressing myself.*

*I have moderate problems washing or dressing myself.*

*I have severe problems washing or dressing myself.*

*I am unable to wash or dress myself.*

Usual activities (e.g. work, study, housework, family, or leisure activities):

*I have no problems doing my usual activities.*

*I have slight problems doing my usual activities.*

*I have moderate problems doing my usual activities.*

*I have severe problems doing my usual activities.*

*I am unable to do my usual activities.*

Pain/discomfort:

*I have no pain or discomfort.*

*I have slight pain or discomfort.*

*I have moderate pain or discomfort.*

*I have severe pain or discomfort.*

*I have extreme pain or discomfort.*

Anxiety/depression:

*I am not anxious or depressed.*

*I am slightly anxious or depressed.*

*I am moderately anxious or depressed.*

*I am severely anxious or depressed.*

*I am extremely anxious or depressed.*

To help people say how good or bad their health is, we would like you to think about a scale (rather like a thermometer) on which the best state you can imagine is marked 100, and the worst state you can imagine is marked 0. We would like you to indicate how good or bad your own health is today, in your opinion. On a scale of 0–100 (100 being the best imaginable), how is your health today? Please enter the number in the box.

#### Stoma Quality of Life Scale

Please read each statement below and decide the way it applies to you. Some questions may seem to be more important to you than others; however, try to answer all questions to the best of your ability.

Rate your overall satisfaction with your life in general right now, again on a scale of 0–100 with 0 being totally unsatisfied and 100 being totally satisfied.

Rate your overall satisfaction with your life in general during the last month, again on a scale of 0–100 with 0 being totally unsatisfied and 100 being totally satisfied.

For each of the following questions, please choose the answer that best corresponds to you (all answers are completely anonymous and confidential).

*Never, seldom, occasionally, frequently, always, and not relevant*

I am able to participate in hobbies I enjoy.

I am able to go out with my friends.

My stoma interferes with my ability to work or attend college/university/school.

I worry about travelling because of my stoma.

I enjoy sexual activity.

I feel attractive.

My sexual partner is bothered by my stoma.

It bothers me if others are aware I have a stoma.

I worry about a lack of privacy when I need to empty my pouch.

I feel comfortable in my clothing.

I am satisfied with the foods I eat.

I have financial concerns regarding my stoma supplies.

I have problems with odour.

I am able to share my feelings and concerns about my stoma with a family member or friend.

I am embarrassed by gas (noises or rapid filling of bag).

I worry my stoma appliance will leak.

I am bothered by skin irritation around the stoma.

Social situations make me anxious.

I can perform the same household and family duties.

#### Body image

In this questionnaire, you will be asked how you feel about your appearance and about any changes that may have resulted from your disease or treatment. Please read each item carefully and choose the reply which comes closest to the way you have been feeling about yourself during the past week.

*Not at all, a little, quite a bit, and very much*

Have you been feeling self-conscious about your appearance?

Have you felt less physically attractive as a result of your disease or treatment?

Have you been dissatisfied with your appearance when dressed?

Have you been feeling less feminine/masculine as a result of your disease or treatment?

Do you find it difficult to look at yourself naked?

Have you been feeling less sexually attractive as a result of your disease or treatment?

Did you avoid people because of the way you feel about your appearance?

Have you been feeling the treatment has left your body less whole?

Have you felt dissatisfied with your body?

Have you been dissatisfied about the appearance of your scar(s)?

#### Activity and functioning

Please choose up to 5 important activities that you are having trouble with and/or are unable to perform at the moment as a result of the hernia at your stoma site (e.g. putting on socks, swing a golf club).

For each activity you have chosen, please use the 0 (unable to perform an activity at all) to 10 (able to perform the activity the same as before) scale to let us know how hard you are finding that activity.

Note: Only choose activities you are having trouble with; this may mean you only choose 2 or 3 activities. If so, leave the remaining questions blank.

#### Experiences in exercises

The following sentences refer to your overall experiences in exercise as opposed to any particular situation. Using the 1–5 scale below, please indicate the extent to which you agree with these statements.

*I don’t agree at all, I agree a little bit, I somewhat agree, I agree a lot, I completely agree, and not applicable*

I feel I have to make a lot of progress in relation to the goal I want to achieve.

The way I exercise is in agreement with my choices and interests.

I feel I perform successfully the activities in my exercise programme.

My relationships with the people I exercise with are very friendly.

I feel the way I exercise is the way I want to.

I feel exercise is an activity I do very well.

I feel I have excellent communications with the people I exercise with.

I feel the way I exercise is a true expression of who I am.

I am able to meet the demands of the exercise I have been given.

My relationships with the people I exercise with are close.

I feel I have the opportunity to make choices with regard to the way I exercise.

#### Confidence to exercise

For each of the following questions, please choose the number that corresponds to your confidence that you can do the tasks regularly at the present time from 0 (not at all confident) to 10 (totally confident).

How confident are you that you can do gentle exercises to strengthen your abdominal muscles?

How confident are you that you can do aerobic exercises such as walking and cycling?

How confident are you that you can exercise without it causing problems with your stoma?

How confident are you that you can exercise without it causing problems with your parastomal hernia?

Engaging and activating my abdominal and pelvic floor muscles when lifting something:

*Strongly agree, tend to agree, tend to disagree, and strongly disagree*

Is something I do automatically.

Is something I do without having to consciously remember.

Is something I do without thinking.

Is something I start to do before I realise I’m doing it.

#### Parastomal hernia

How soon after surgery did you notice your bulge/hernia?

- *Less than 3 months/3–6 months/6–12 months/12–24 months/more than 24 months*

Do you get pain associated with your bulge/hernia?

- *Yes/no*
- If yes, how bad is the pain on a scale of 1–10?

Does your pain affect any of the following? Select all that apply

- *Being active or participating in your hobbies/completing your day-to-day activities/lifting items you find heavy/doing your job*

What size do you currently consider your bulge/hernia to be?

- *Very small/small/medium/large/very large*

Is your bulge/PSH larger than 5 cm in diameter (a tennis ball is around 6 cm)?

- *Yes/no*

Do you use any of the following to help manage your bulge/hernia? Please select all that apply:

- *Support garments/exercises/dietary management or restriction/others*

On a scale of 0–10, how well do you feel you are managing your bulge/hernia at the moment?

- *0 being not managed at all; 10 being very well managed*

How do you feel about your body image in relation to your parastomal bulge?

- *0 (not at all happy) to 10 (completely happy)*

Does your bulge/hernia affect any of the following? Please select all that apply.

- *Your stoma output/your bag adherence/other bag issues/the food you eat/others*

Have you ever considered a surgical repair for your bulge/hernia?

- *Yes/no*

Are you currently considering a surgical repair for your parastomal bulge?

- *Yes/no*

#### Your physical activity

In the past week, how many days have you done physical activity indoors (e.g. online exercise class, self-workout, yoga)?

- *0, 1, 2, 3, 4, 5, 6, and 7*

What was the usual length of your activity (in minutes)?

In the past week, how many days have you done physical activity outdoors (e.g. walk, run, cycle)?

- *0, 1, 2, 3, 4, 5, 6, and 7*

What was the usual length of the activity (in minutes)?

## Appendix 4

**Table 7:** Missing data rate for the outcome measures

**Outcome measure**	**Missing data rate %**		
	**Single-arm trial (** ***n*** ** = 8)**	**Feasibility RCT**	
		**Control (** ***n*** ** = 4)**	**Intervention (** ***n*** ** = 4)**
EQ5D Descriptive Score	12.5	0	0
EQ5D VAS	0	0	0
Stoma-QOL now	0	25	0
Stoma-QOL past month	0	25	25
Stoma-QOL work/social functioning	37.5	100	50
Stoma-QOL sexuality/body image	37.5	75	50
Stoma-QOL stoma function	0	0	0
Stoma-QOL financial concerns	0	0	0
Stoma-QOL skin irritation	0	0	0
Self-efficacy	0	25	0
Physical activity	0	0	0
Do you have pain associated with your bulge/hernia?	0	0	0
What size do you consider your bulge/hernia to be?	0	0	0
Is your bulge/hernia larger than 5 cm in diameter?	0	0	0
How do you feel about managing your bulge/hernia?	0	0	25
How do you feel about your body image in relation to your bulge/hernia?	0	0	0
How you ever considered surgical repair?	12.5	50	0
Are you currently considering surgical repair?	0	25	0

## Appendix 5

### Results of outcomes

**Table 8:** Results of the paired *t*-tests showing the mean difference between baseline and follow-up in the single-arm trial are presented below

**Scales (range)**	**Number**	**Baseline mean (SD)**	**Follow-up mean (SD)**	**Mean difference (SD)**	**95%CI**
EQ5D Descriptive Score (− 1–1)	7	0.55 (0.40)	0.62 (0.33)	0.07 (.23)	− 0.13; 0.28
EQ5D VAS (0–100)	8	60.00 (27.26)	71.00 (24.69)	11.00 (11.81)	1.13; 20.87
Stoma-QOL now (0–100)	8	65.63 (32.12)	65.00 (30.36)	− 0.63 (11.16)	− 9.96; 8.71
Stoma-QOL past month (0–100)	8	61.88 (32.84)	63.75 (29.97)	1.88 (11.00)	− 7.32; 11.07
Stoma-QOL work/social function (0–100)	5	59.17 (28.78)	67.50 (23.46)	8.33 (13.50)	− 8.43; 25.10
Stoma-QOL sexuality/body image (0–100)	5	70.00 (23.72)	65.00 (21.51)	− 5.00 (7.07)	− 13.78; 3.78
Stoma-QOL stoma function (0–100)	8	68.23 (26.06)	65.63 (17.64)	− 2.60 (13.90)	− 14.22; 9.02
Stoma-QOL financial concerns (0–100)	8	84.38 (35.20)	100.00 (0)	15.63 (35.20)	− 13.80; 45.05
Stoma-QOL skin irritation (0–100)	8	65.63 (18.60)	59.38 (29.69)	− 6.25 (17.68)	− 21.03; 8.53
Body Image Scale (0–30)	8	12.63 (10.56)	13.88 (9.00)	1.25 (3.28)	− 1.50; 4.00
Self-efficacy (1–10)	8	6.41 (3.32)	7.63 (2.57)	1.22 (1.47)	− 0.01; 2.45
Patient-Specific Functional Scale (0–10)	7	2.53 (1.87)	3.88 (2.61)	1.35 (1.31)	0.14; 2.56
Physical activity	8	244.38 (233.72)	179.00 (165.27)	− 65.38 (173.90)	− 210.76; 80.01
Ability to manage hernia	8	5.25 (2.87)	6.13 (3.72)	0.88 (1.55)	− 0.42; 2.17
Body image in relation to bulge/hernia	8	2.75 (3.45)	3.00 (3.34)	0.25 (1.49)	− 0.99; 1.49

**Table 9:** Results of the independent *t*-tests of the mean change scores from baseline to follow-up of the control and intervention groups

**Scales (range)**	**Group**	**Number**	**Baseline mean (SD)**	**Follow-up mean (SD)**	**Mean change (SD)**	**Mean difference (SD)**	**95%CI**
EQ5D Descriptive Score (− 1–1)	Control	4	0.68 (0.09)	0.78 (0.19)	0.10 (0.17)	0.13 (0.10)	− 0.13; 0.38
	Intervention	4	0.80 (0.06)	0.77 (0.80)	− 0.03 (0.12)		
EQ5D VAS (0–100)	Control	4	65.00 (17.80)	83.13 (12.81)	18.13 (13.44)	18.13 (16.74)	− 22.75; 59.10
	Intervention	4	85.00 (12.68)	85.00 (23.45)	0 (30.67)		
Stoma-QOL now (0–100)	Control	3	66.67 (23.09)	65.83 (27.42)	− 0.83 (25.54)	− 5.58 (12.57)	− 37.90; 26.74
	Intervention	4	89.00 (8.60)	93.75 (6.29)	4.75 (4.11)		
Stoma-QOL past month (0–100)	Control	3	71.67 (20.21)	65.83 (27.42)	− 5.83 (17.01)	− 18.83 (10.43)	− 47.80; 10.14
	Intervention	3	82.00 (10.82)	95.00 (5.00)	13.00 (6.08)		
Stoma-QOL work/social function (0–100)	Control	0	–	–	–	–	–
	Intervention	2	87.50 (5.89)	75.00 (5.89)	− 12.50 (0)		
Stoma-QOL sexuality/body image (0–100)	Control	1	30.00	35.00	5.00	12.50 (4.33)	− 42.52; 67.52
	Intervention	2	82.50 (10.60)	75.00 (14.14)	− 7.50 (3.54)		
Stoma-QOL stoma function (0–100)	Control	4	46.88 (19.06)	42.71 (22.41)	− 4.17 (5.89)	− 22.92 (7.12)	− 40.33; − 5.51
	Intervention	4	55.21 (17.47)	73.96 (18.44)	18.75 (12.96)		
Stoma-QOL financial concerns (0–100)	Control	4	87.50 (25.00)	87.50 (25.00)	0 (0)	–	–
	Intervention	4	100.00	100.00	0 (0)		
Stoma-QOL skin irritation (0–100)	Control	4	50.00 (20.41)	25.00 (20.41)	− 25.00 (35.36)	− 43.75 (21.35)	− 95.99; 10.28
	Intervention	4	43.75 (31.46)	62.50 (25.00)	18.75 (23.94)		
Body Image Scale (0–30)	Control	4	10.75 (6.65)	11.25 (5.32)	0.50 (1.73)	2.75 (2.85)	− 4.23; 9.73
	Intervention	4	7.00 (4.16)	4.75 (2.22)	− 2.25 (5.44)		
Self-efficacy (1–10)	Control	3	3.33 (1.33)	4.50 (1.80)	1.17 (1.92)	− 0.02 (1.05)	− 2.72; 2.68
	Intervention	4	8.38 (0.63)	9.56 (0.55)	1.19 (0.83)		
Patient-Specific Functional Scale (0–10)	Control	3	3.21 (0.66)	2.94 (0.42)	− 0.27 (0.69)	1.57 (0.80)	− 1.86; 4.99
	Intervention	1	5.33	3.50	− 1.83		
Physical activity	Control	4	286.00 (452.79)	297.50 (259.28)	11.50 (406.87)	322.75 (439.25)	− 752.04; 1397.54
	Intervention	4	731.25 (598.67)	420.00 (519.47)	− 311.25 (778.59)		
Ability to manage bulge/hernia	Control	4	5.75 (3.20)	4.25 (1.71)	− 1.50 (1.73)	− 4.17 (1.80)	− 8.79; 0.45
	Intervention	3	5.33 (1.16)	8.00 (2.00)	2.67 (3.06)		
Body image in relation to bulge/hernia	Control	4	2.75 (2.50)	1.00 (1.41)	− 1.75 (1.89)	− 2.50 (2.94)	− 9.69; 4.67
	Intervention	4	4.75 (4.27)	5.50 (1.73)	0.75 (5.56)

**Table 10:** Descriptive statistics for questions related to bulge/hernia are presented below

**Outcome measure**	**Single arm trial**		**Feasibility RCT**			
	**Baseline, ** ***n*** ** (%)**	**Follow-up, ** ***n*** ** (%)**	**Control**		**Intervention**	
			**Baseline, ** ***n*** ** (%)**	**Follow-up, ** ***n*** ** (%)**	**Baseline, ** ***n*** ** (%)**	**Follow-up, ** ***n*** ** (%)**
Do you have pain associated with your bulge/hernia?						
Yes	7 (87.5)	8 (100)	2 (50.0)	1 (25.0)	3 (75.0)	1 (25.0)
No	1 (12.5)	0	2 (50.0)	3 (75.0)	1 (25.0)	3 (75.0)
If yes, what is your pain score (mean (SD))	4.71 (2.87)	4.88 (3.09)	2.00 (1.41)	7.00	1.33 (0.58)	1.00
Does your pain affect any of the following?						
Being active or participating in your hobbies	6 (75.0)	6 (75.0)	0	1 (25.0)	0	0
Completing your day-to-day activities	6 (75.0)	6 (75.0)	0	1 (25.0)	0	0
Lifting items you find heavy	6 (75.0)	7 (87.5)	1 (25.0)	1 (25.0)	2 (50.0)	0
Doing your job	2 (25.0)	1 (12.5)	0	0	0	0
Others	0	0	1 (25.0)	0	0	0
What size do you consider your bulge/hernia to be?						
Small	2 (25.0)	1 (12.5)	0	0	1 (25.0)	2 (50.0)
Medium	3 (37.5)	3 (37.5)	3 (75.0)	0	2 (50.0)	2 (50.0)
Large	2 (25.0)	3 (37.5)	1 (25.0)	3 (75.0)	0	0
Very large	1 (12.5)	1 (12.5)	0	1 (25.0)	1 (25.0)	0
Is your bulge/hernia larger than 5 cm in diameter?						
Yes	7 (87.5)	6 (75.0)	3 (75.0)	4 (100.0)	2 (50.0)	2 (50.0)
No	1 (12.5)	2 (25.0)	1 (25.0)	0	2 (50.0)	2 (50.0)
Do you use any of the following to help manage your bulge/hernia?						
Support garments	8 (100)	8 (100)	3 (75.0)	3 (75.0)	4 (100.0)	4 (100.0)
Exercises	5 (62.5)	6 (75.0)	0	1 (25.0)	0	4 (100.0)
Dietary management or restriction	3 (37.5)	4 (50.0)	0	0	0	0
Others	0	0	0	0	0	0
Does your bulge/hernia affect any of the following?						
Your stoma output	2 (25.0)	3 (37.5)	0	1 (25.0)	2 (50.0)	0
Your bag adherence	1 (12.5)	2 (25.0)	3 (75.0)	3 (75.0)	1 (25.0)	2 (50.0)
Other bag issues	0	1 (12.5)	2 (50.0)	0	1 (25.0)	1 (25.0)
The food you eat	3 (37.5)	3 (37.5)	1 (25.0)	0	0	0
Others	0	1 (12.5)	0	0	1 (25.0)	0
Have you ever considered surgical repair?						
Yes	3 (37.5)	3 (37.5)	2 (50.0)	1 (25.0)	2 (50.0)	3 (75.0)
No	5 (62.5)	4 (50.0)	2 (50.0)	1 (25.0)	2 (50.0)	1 (25.0)
Are you currently considering surgical repair?						
Yes	2 (25.0)	3 (37.5)	1 (25.0)	1 (25.0)	1 (25.0)	2 (50.0)
No	6 (75.0)	5 (62.5)	3 (75.0)	2 (50.0)	3 (75.0)	2 (50.0)
